# Incremental benefit of booster vaccinations for COVID-19 in the United Kingdom

**DOI:** 10.1016/j.lanepe.2023.100790

**Published:** 2023-11-17

**Authors:** Benjamin J. Cowling, Sheena G. Sullivan

**Affiliations:** a WHO Collaborating Centre for Infectious Disease Epidemiology and Control, School of Public Health, The University of Hong Kong, Hong Kong Special Administrative Region, China; b Laboratory of Data Discovery for Health Limited, Hong Kong Science and Technology Park, New Territories, Hong Kong Special Administrative Region, China; c WHO Collaborating Centre for Reference and Research on Influenza, Royal Melbourne Hospital, and Department of Infectious Diseases, University of Melbourne, at the Peter Doherty Institute for Infection and Immunity, Melbourne, Victoria, Australia; d Department of Epidemiology, University of California, Los Angeles, California, USA

It has been estimated that COVID-19 vaccines had saved hundreds of thousands of lives in the European Union by the end of 2021.^1^ From 2022, additional 'booster' doses of COVID-19 vaccine have been recommended on an annual basis in the United Kingdom and other countries, particularly for people at higher risk of severe COVID-19, including older adults and people with underlying medical conditions. Previous studies in the UK have shown that booster doses with the original monovalent formulation reduced the risk of hospitalization with Omicron BA.1,^2^ and Omicron BA.4/BA.5.^3^ In the autumn of 2022, COVID-19 vaccines were updated to include an Omicron strain as well as the original vaccine strain, with the UK choosing to use a bivalent vaccine with Omicron BA.1 and other countries such as the United States choosing to use a bivalent vaccine with Omicron BA.4/BA.5.^4^

In this issue of *The Lancet Regional Health—Europe*, Kirsebom et al. linked multiple electronic health records in England to estimate COVID-19 vaccine effectiveness (VE). They used a test-negative study design where patients with positive tests for COVID-19 between December 2022 and April 2023 were included as cases and those testing negative for COVID-19 were controls, limited to patients with primary diagnosis codes for acute respiratory illness. They included 3881 cases, stratified in analyses by subvariant, and 187,348 test-negative controls. They report that bivalent COVID-19 vaccines were effective in preventing COVID-19 hospitalizations with incremental VE estimates of 48.0% (95% C.I.; 38.5–56.0%), 29.7% (95% C.I.; 7.5–46.6%) and 52.7% (95% C.I.; 24.6–70.4%), against BQ.1, CH.1.1 and XBB.1.5, respectively, at 5–9 weeks post vaccination.^5^ Estimates of incremental VE declined over time.

These observations support their earlier report that booster doses of bivalent vaccine were effective in preventing COVID-19 hospitalizations between September 5, 2022 and February 5, 2023 when various Omicron subvariants were circulating.^4^ Together, these reports support the use of bivalent vaccines for protection against severe COVID-19. Importantly, this new study provides evidence that the bivalent vaccine provides cross-protection against antigenically-distinct circulating Omicron subvariants. Two important methodological features of this study deserve further consideration, the interpretation of “incremental VE” and the challenges of analyses which use electronic health records.

The term “vaccine effectiveness” is a causal estimate of the degree of protection provided to vaccinated individuals by vaccination.^6^ In studies of the impact of COVID-19 boosters, the choice of comparison group matters for interpretation. For example, individuals who have recently received a fourth vaccine dose could be compared to individuals who have never been vaccinated, or to individuals eligible for a fourth dose who have chosen not to receive it. It could be argued that the latter group is the most appropriate comparison group, and that the VE could be referred to as “incremental VE”. Looking to the future, there will be increasing permutations of dose number, recency and type of vaccine, and analyses of VE may need to be simplified to assess the incremental VE of recently-administered boosters in a similar way to the approach for annual influenza VE assessment.^7^ Comparisons with never-vaccinated individuals is becoming increasingly selective and arguably of less public health value, noting that almost all vaccine doses now administered are booster doses.

The approach used by Kirsebom et al. involved data linkage of multiple electronic medical records,^5^ which could have several potential limitations. First, data linkage is not perfect and even where a unique identifier may be available, data linkage errors can occur. Whether this results in under or overestimation of VE may not be possible to predict.^8^ Moreover, opportunistic use of electronic medical records can mean that cases and non-cases are not derived from the same source population. This problem is avoided in prospective test-negative studies through use of a common clinical case definition. In analyses of electronic medical records such as the study by Kirsebom et al., this is approximated by restricting the sample to patients with common admission and/or discharge codes.^9^ However, this approach may not work for all such studies, particularly when the reason for admission is updated post-discharge and informed by testing that was performed in hospital.

In conclusion, Kirsebom et al. have provided timely estimates of VE, confirming the continued impact of booster vaccination campaigns even against evolving Omicron subvariants. A further strain update has been recommended in COVID-19 boosters used in the United Kingdom and elsewhere, with the XBB.1.5 strain in a monovalent formulation for the 2023/2024 northern hemisphere winter. Given that Kirsebom et al. only investigated the durability of protection for up to at most 6 months after the booster dose, further studies are needed to examine durability of protection over a longer period to provide relevant data for annual booster programs. Monitoring of the VE of COVID-19 boosters should be continued as an important routine public health activity.

## Contributors

Wrote first draft: BJC. Revised manuscript for important intellectual content: BJC and SGS. Both authors have seen the final version and approve of publication.

## Declaration of interests

BJC has received honoraria from AstraZeneca, Fosun Pharma, GSK, Haleon, Moderna, Novavax, Pfizer, Roche, and Sanofi Pasteur. SGS has received honoraria from CSL, Evohealth, Moderna, and Pfizer.
